# Blood levels of neurotransmitters in Yusho patients: An approach via the descending pain inhibitory pathway for persistent sensory disturbance

**DOI:** 10.1111/1346-8138.17689

**Published:** 2025-03-03

**Authors:** Miwa Ashida, Naoya Murayama, Yoshiyuki Kamio, Mariko Yozaki, Yutaka Kuwatsuka, Takeshi Nakahara, Hiroyuki Murota

**Affiliations:** ^1^ Department of Dermatology Nagasaki University Graduate School of Biomedical Sciences Nagasaki Japan; ^2^ Sasebo City General Hospital Sasebo‐shi Nagasaki Japan; ^3^ Honda Dermatology Allergy Clinic Omura‐shi Nagasaki Japan; ^4^ Research and Clinical Center for Yusho and Dioxin Kyushu University Hospital Fukuoka Japan

**Keywords:** descending pain inhibitory pathway, dioxins, neurotransmitter, sensory disorders, Yusho disease

## Abstract

Yusho, a dioxin poisoning incident in Japan, has resulted in patients experiencing persistent symptoms, including sensory disturbances, decades after the initial exposure. This study investigated the potential involvement of the descending pain inhibitory system in Yusho patients. Serum serotonin, dopamine, and norepinephrine levels were measured in 29 Yusho patients and 29 age‐matched healthy controls. No significant differences in these neurotransmitters were observed between the two groups. However, weak correlations were found between polychlorinated biphenyl levels and dopamine (*r* = 0.4310, *p* = 0.0315) in Yusho patients. This study provides new insights into the pathophysiology of cutaneous sensory disorders and highlights the need for further research to clarify the long‐term effects of dioxin exposure on Yusho patients.

## 1 INTRODUCTION

In 1968, the Yusho incident occurred in western Japan due to ingestion of dioxin‐contaminated rice oil.^1^ Subsequent investigations revealed that the contaminated oil contained a mixture of dioxins including polychlorinated biphenyl (PCB), polychlorinated quaterphenyl (PCQ), and polychlorinated dibenzofuran (PCDF). The outbreak was concentrated in Nagasaki Prefecture, where our hospitals are based, prompting us to continue research with a focus on innate immune responses.^2^, ^3^ Fifty‐six years after the incident, elevated dioxin levels remain in the patients' serum.^4^ The number of patients presenting with dioxin‐induced skin symptoms, such as acneiform eruptions, skin and mucosal pigmentation, and meibomian gland hypersecretion, has decreased. However, many patients still complain of sensory abnormalities that are not severe but intangible, such as numbness, paresthesia, and abnormal tactile and thermal sensations of the extremities, which are 1.3–1.5 times higher than those in healthy subjects.^5^ There have been limited reports on the evaluation of abnormal cutaneous sensations, including peripheral neuropathy associated with dioxin exposure, particularly in Yusho.^5^

Recently, regulation of the interleukin (IL)‐33/IL‐37 axis by aryl hydrocarbon receptors (AhR), a receptor for dioxins, has been applied in the treatment of atopic dermatitis and psoriasis.^6^, ^7^ The research conducted by Akahoshi et al. demonstrated that AhR activation influences tyrosine hydroxylase expression, an enzyme crucial for the synthesis of catecholaminergic neurotransmitters.^8^ Catecholamines, such as serotonin, norepinephrine, and dopamine, are neurotransmitters that regulate the descending pain inhibitory pathway. This system comprises serotonergic and noradrenergic pathways that primarily descend from the brainstem to the dorsal horn of the spinal cord, and functions as a defense mechanism that suppresses pain by binding neurotransmitters to pain receptors in the spinal dorsal horn.^9^ Individuals with diminished levels of catecholamines exhibit increased susceptibility to pain and chronic pain.^10^ We hypothesized that the aberrant AhR function in patients with Yusho may influence the production of catecholamines and induce dysfunction in the descending pain inhibitory system, resulting in the sensory disturbances reported by patients. If a significant association is established between the aberrant AhR function the descending pain inhibitory system, it could be the basis of a therapeutic approach targeting the descending inhibitory system in Yusho patients. Therefore, we examined the levels of serotonin, dopamine, and norepinephrine in Yusho patients which play key roles in this system.

## 2 METHODS

All data are presented in the Table S1.

### 2.1 Materials

A total of 58 individuals, comprising 29 patients diagnosed with Yusho, and 29 age‐matched healthy subjects were recruited for the study. Participants who underwent dioxin level measurements and did not fulfill the Yusho criteria were designated healthy controls. The mean (± standard deviation) age of the Yusho patients was 72.0 ± 8.6 years, while that of the controls was 67.9 ± 11.0 years. Blood samples were obtained from participants during their annual medical examinations, conducted in Nagasaki Prefecture between 2005 and 2009.

### 2.2 Measurement of serum serotonin, dopamine, and norepinephrine

Frozen blood samples collected at the time of the annual medical examinations were used for measurements. A Serotonin enzyme‐linked immunosorbent assay (ELISA) Kit (Abcam, ab133053), Dopamine ELISA Kit (Abcam, ab285238), and Norepinephrine ELISA Kit (Abcam, ab287789) were employed to quantify serotonin, dopamine, and norepinephrine levels in the serum samples, respectively.

### 2.3 Correlation with dioxin concentrations

Blood concentrations of PCB (ppb), PCQ (ppb), and PCDF (pg/g lipids) were obtained from a database of Yusho patient measurements, which had been assessed at each annual examination using high‐resolution gas chromatography/high‐resolution mass spectrometry.^11^

### 2.4 Statistical analysis

Statistical analysis was performed using the GraphPad Prism software, version10 (GraphPad Software). The Mann–Whitney *U* test was used to compare serum serotonin, dopamine, and norepinephrine levels between the Yusho patients and controls. Spearman's rank correlation analysis was used to evaluate the relationships between PCB, PCQ, and PCDF levels and serum serotonin, dopamine, and norepinephrine levels. Multiple linear regression analysis was performed to identify the factors influencing age. Outliers were not excluded from analysis. Statistical significance was set at *p* < 0.05.

## 3 RESULTS

### 3.1 Measurement of serum serotonin, dopamine, and norepinephrine

**FIGURE 1:**
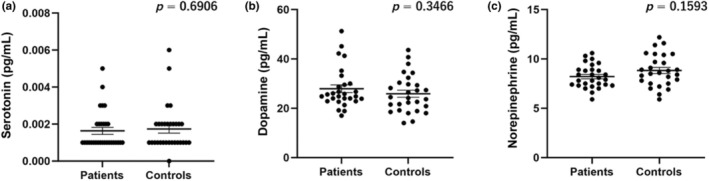
Comparison of serum (a) serotonin, (b) dopamine, and (c) norepinephrine levels between Yusho patients and healthy controls. Serum serotonin, dopamine, and norepinephrine concentrations were not significantly different between Yusho patients and controls. Error bars represent the standard deviation of the mean.

Figure 1 illustrates the comparison of serum serotonin, dopamine, and norepinephrine levels between the Yusho patients and healthy controls. No significant differences were observed between the two groups in terms of serotonin (*p* = 0.6906), dopamine (*p* = 0.3466), or norepinephrine (*p* = 0.1593).

### 3.2 Correlation with dioxin concentrations

**FIGURE 2:**
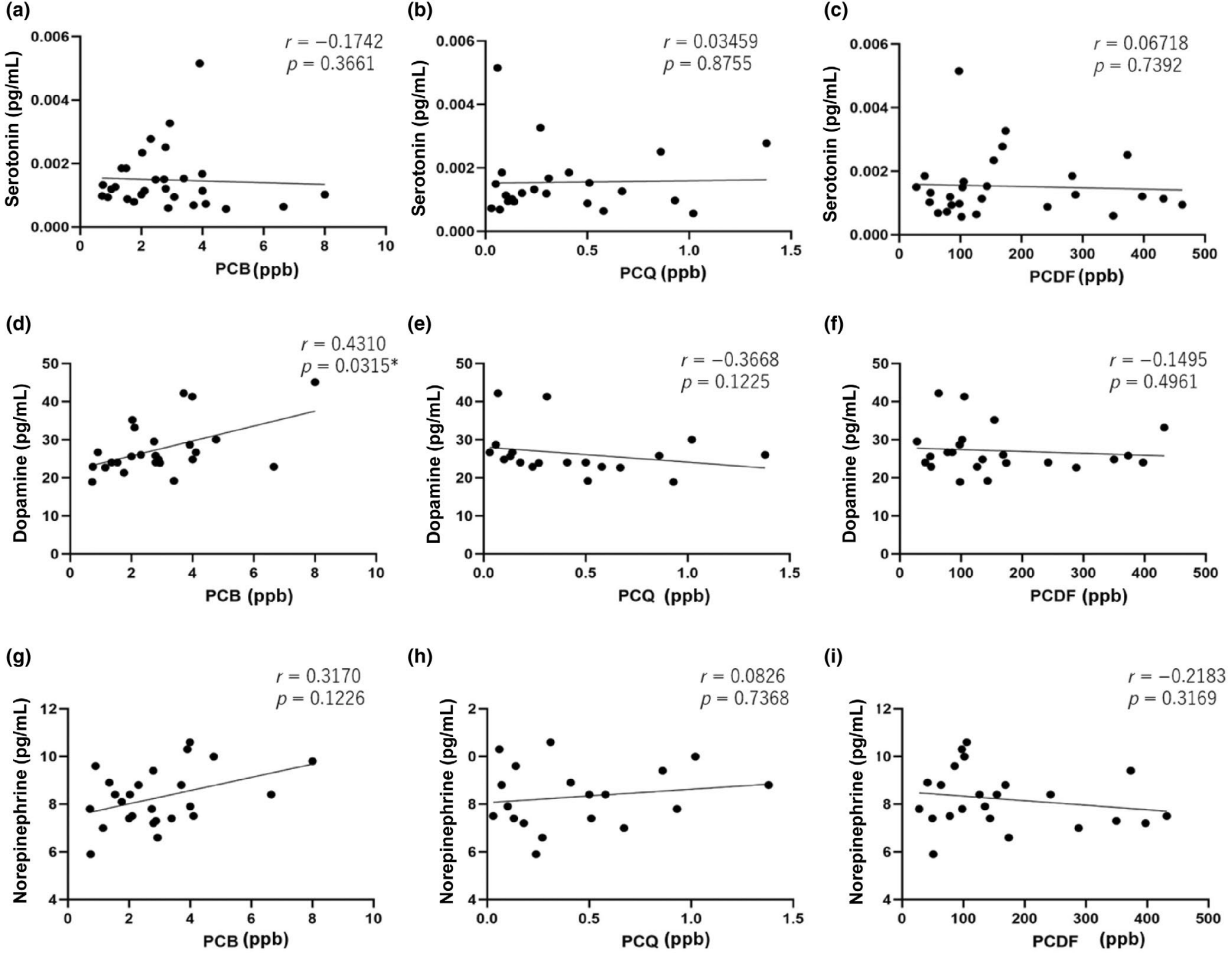
Depiction of the relationship between serum polychlorinated biphenyl (PCB), polychlorinated quaterphenyl (PCQ), and polychlorinated dibenzofuran (PCDF) levels and (a–c) serotonin (d–f) dopamine, and (g–i) norepinephrine levels in Yusho patients. A weak correlation was observed between PCB levels and serum dopamine levels. No correlation was found between serotonin or norepinephrine levels and PCB, PCQ, or PCDF. *p < 0.05.

Figure 2 depicts the serum serotonin, dopamine, and norepinephrine levels and dioxin concentrations in the Yusho group. There were no correlations between PCB, PCQ, PCDF, and serotonin levels (*r* = −0.1742, *p* = 0.3661; *r* = 0.03459, *p* = 0.8755; *r* = 0.06718, *p* = 0.7392, respectively). Although no correlation was observed between PCQ, PCDF and dopamine levels (*r* = −0.03668, *p* = 0.1225; *r* = −0.1495, *p* = 0.4961, respectively), a weak correlation was detected between PCB and dopamine levels (*r* = 0.4310, *p* = 0.0315). No correlation was found between PCB, PCQ, PCDF and norepinephrine levels (*r* = 0.3170, *p* = 0.1226, *r* = 0.0826, *p* = 0.7368, *r* = −0.2183, *p* = 0.3169, respectively).

## 4 DISCUSSION

This study focused on abnormalities in skin sensation observed in Yusho patients, a continuation of several related investigations. In preliminary experiments using a thermometer, one patient with the lowest cold‐sensation threshold exhibited the highest concentrations of PCB, PCQ, and PCDF (Figure S1). However, the concentration of artemin, a neurotrophic factor involved in thermal hyperalgesia of the skin,^12^ did not differ significantly between Yusho patients and healthy controls (Figure S2).

Serotonin is a neurotransmitter that regulates dopamine and norepinephrine levels in the brain and contributes to the maintenance of mental stability. Dopamine and its precursor, norepinephrine, play crucial roles in the central nervous system by influencing motivation, memory, fear, and the sleep–wake cycle. Serotonin and norepinephrine are also involved in pain modulation, activation of the descending pain inhibitory system, and facilitation of pain relief.^13^ Dysfunction of this system, due to factors such as psychological stress, anxiety, or depression, may lead to hyperalgesia.

Studies on dopamine and dioxin have demonstrated that dopamine concentrations were reduced in the cellular structures and brains of rats exposed to PCBs.^14^ Similarly, lower levels of homovanillic acid (HVA), a dopamine metabolite, were observed in individuals with higher PCB exposure.^15^ These findings suggest that PCB exposure may be associated with depression. Based on this, we hypothesized that patients may experience decreased dopamine levels due to dioxin exposure, potentially leading to impaired function of the descending pain inhibitory system and increased pain sensitivity.

However, our results showed that patients with elevated blood PCB levels had higher serum dopamine levels. Dioxins accumulate in healthy individuals in an age‐dependent manner owing to environmental exposure,^16^ an effect not fully accounted for in the multiple regression analysis. It is possible that patients with elevated dioxin blood concentrations may exhibit overactivity of the descending pain inhibitory system, possibly resulting in hyposensitivity and impaired heat or pain sensation. Some reports suggest that serotonin and noradrenaline suppress itching through the descending inhibitory mechanism.^17^, ^18^ Although we did not evaluate itching in this study, we believe that the investigation of neurotransmitters is a useful exploration of sensory disturbances in patients with Yusho, since itching and pain are intricately related to each other.

Limitations of the present study include the lower sensitivity of serotonin and norepinephrine measurements and the absence of a quantitative assessment of the degree of chronic neuropathy. Blood concentrations of dopamine and norepinephrine are influenced by factors such as age, blood pressure, blood glucose levels, psychological stress, physical activity, and postural changes. Urinary HVA, a metabolite of dopamine, could provide additional validation of our findings.

To the best of our knowledge, this is the first study to investigate the catecholaminergic neurotransmitter‐activated descending pain system in response to sensory disturbances in Yusho. Clarifying the pathophysiology of this pathway will contribute to a more comprehensive understanding of cutaneous sensory disorders.

## CONFLICT OF INTEREST STATEMENT

None declared.

## ETHICS STATEMENT

This study was approved by the Nagasaki University Hospital Clinical Research Ethics Committee (approval no. 14062379‐6, 19/4/2022). All participants provided informed consent prior to participating in the study.

## Supporting information


Appendix S1.
