# Supplementary material for: Blood levels of neurotransmitters in Yusho patients: An approach via the descending pain inhibitory pathway for persistent sensory disturbance
Source: J Dermatol. 2025 Mar 3;52(5):934–8. doi: 10.1111/1346-8138.17689 (PMC12056279; doi:10.1111/1346-8138.17689)
Supplement: Supplementary file 1 — Appendix S1. [file JDE-52-934-s001.docx]

| **Serotonin** | | **Dopamine** | | **Norepinephrine** | | **Dioxins in Patients** | | |
| --- | --- | --- | --- | --- | --- | --- | --- | --- |
| patient | Controls | Patients | Controls | Patients | Controls | PCB | PCQ | PCDF |
| 0.001 | 0.002 | 42.2 | 23.7 | 8.8 | 8.8 | 3.08 | 0.11 | 462.88 |
| 0.002 | 0.001 | 24 | 21.5 | 8.4 | 10.4 | 1.5 | 0.08 | 282.81 |
| 0.001 | 0.005 | 26 | 34.7 | 8.8 | 12.2 | 2.47 | 0.05 | 103.41 |
| 0.001 | 0.001 | 25.8 | 19.2 | 9.4 | 7 | 1.02 | 0.3 | 83.2 |
| 0.001 | 0.003 | 23.9 | 18 | 6.6 | 8.9 | 3.71 | 0.07 | 63.34 |
| 0.001 | 0.001 | 22.7 | 27.2 | 7 | 7.8 | 1.54 | 0.5 | 242.48 |
| 0.003 | 0.002 | 24 | 14.6 | 7.2 | 9.6 | 2.31 | 1.38 | 168.83 |
| 0.003 | 0.002 | 19.2 | 18 | 7.4 | 9.7 | 2.79 | 0.86 | 373.1 |
| 0.003 | 0.001 | 28.7 | 21.7 | 10.3 | 11.4 | 2.93 | 0.27 | 174.03 |
| 0.001 | 0 | 25.6 | 18.9 | 7.4 | 11.6 | 1.15 | 0.67 | 288.12 |
| 0.001 | 0.002 | 41.3 | 40.7 | 10.6 | 8.4 | 2.8 | 0.18 | 397.28 |
| 0.002 | 0.002 | 24.8 | 20.9 | 7.9 |  | 3.39 | 0.51 | 143.59 |
| 0.005 | 0.001 | 26.7 | 18.5 | 7.5 | 10.6 | 3.91 | 0.06 | 97.77 |
| 0.001 | 0.001 | 22.9 | 14 | 8.4 | 7.2 | 2 | 0.13 | 49.71 |
| 0.002 | 0.002 | 30 | 32.4 | 10 | 10.5 | 3.99 | 0.31 | 105.28 |
| 0.001 | 0.001 | 24 | 43.6 | 8.9 | 7.5 | 4 | 0.1 | 135.15 |
| 0.001 | 0.001 | 26.7 | 22.3 | 9.6 | 6.4 | 4.11 | 0.03 | 77.96 |
| 0.001 | 0.001 | 35.2 | 24.5 | 8.4 | 7.9 | 6.65 | 0.58 | 126.27 |
| 0.001 | 0.001 | 29.5 | 29.3 | 7.8 | 5.9 | 4.77 | 1.02 | 101.67 |
| 0.002 | 0.002 | 21.3 | 29.7 | 8.1 | 6.9 | 1.35 | 0.41 | 41.64 |
| 0.001 | 0.001 | 22.9 | 22.6 | 5.9 | 8.2 | 0.9 | 0.14 | 85.66 |
| 0.002 | 0.001 | 33.2 | 34.4 | 7.5 | 8.3 | 2.03 |  | 154.59 |
| 0.002 | 0.002 | 18.9 | 26.5 | 7.8 | 8.9 | 2.74 |  | 27.79 |
| 0.001 | 0.002 | 24.8 | 28.2 | 7.3 | 8.6 | 1.76 |  |  |
| 0.001 | 0.001 | 24.8 | 30.4 | 7 | 8.5 | 0.74 | 0.24 | 50.9 |
| 0.001 | 0.001 | 45.1 | 38 | 9.8 | 10.5 | 2.1 |  | 432.16 |
| 0.001 | 0.001 | 51.3 | 24.2 | 8.9 | 7.5 | 0.72 | 0.93 | 98.28 |
| 0.001 | 0.003 | 17 | 27.4 | 7.3 | 9.1 | 2.88 |  | 349.91 |
| 0.004 | 0.006 |  |  |  |  | 8 |  |  |
| 0.001 | 0.002 |  |  |  |  |  |  |  |

**Sup. Table.** Data used in this study included serotonin, dopamine, and norepinephrine levels in patients and healthy subjects, and dioxin levels in patients.

**
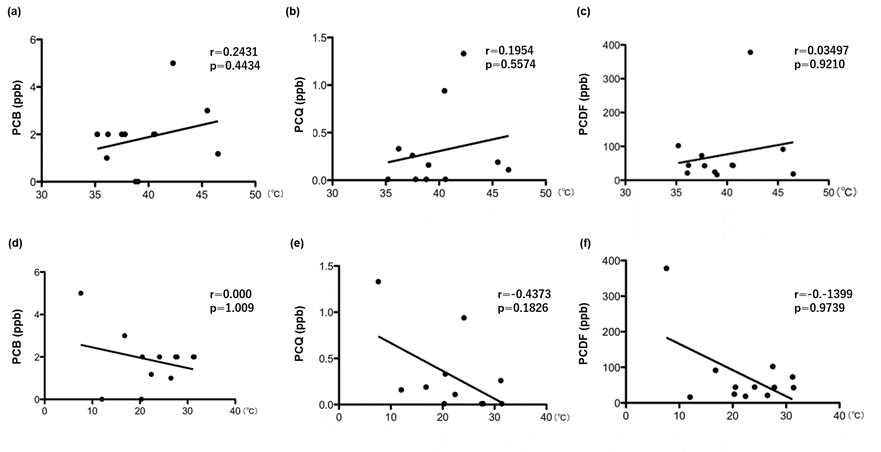
Sup. Figure 1**

Sup. Fig.1. Correlations between serum PCB, PCQ, and PCDF levels and the threshold for sensing heat (a-c) and the threshold for sensing cold (d-f) in Yusho patients. PCB: polychlorinated biphenyl. PCQ: polychlorinated quaterphenyl. PCDF: polychlorinated dibenzofuran.


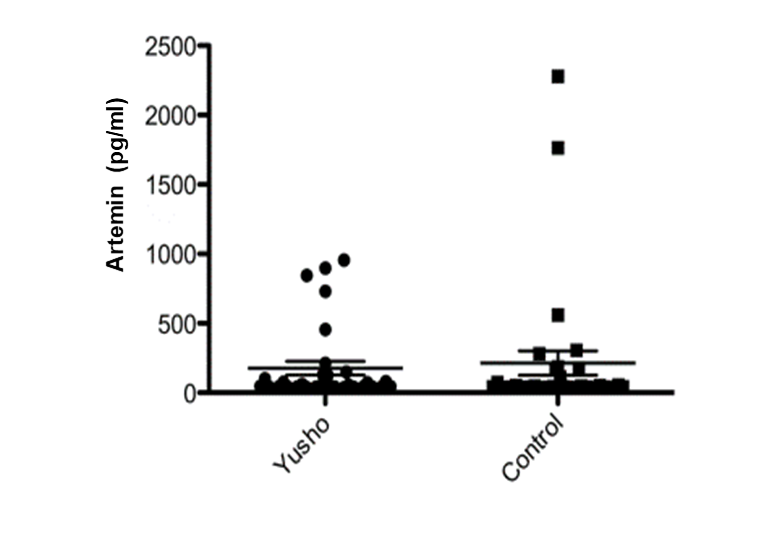
**Sup. Figure 2**

Sup. Fig.2. Comparison of serum artemin levels in Yusho patients and healthy controls.
